# Delayed Diagnosis of Spinal Cord Injury in a Patient With Intellectual Disability: A Case Report

**DOI:** 10.7759/cureus.59588

**Published:** 2024-05-03

**Authors:** Sayaka Ito, Yoshinori Maki, Naoki Hatsuda

**Affiliations:** 1 Neurosurgery, Kohka Public Hospital, Kohka, JPN; 2 Neurosurgery, Hikone Chuo Hospital, Hikone, JPN; 3 Rehabilitation, Hikari Hospital, Otsu, JPN

**Keywords:** spinal cord injuries, intellectual disability (id), rehabilitation, trauma, cervical decompression surgery, neurological deficits

## Abstract

Spinal cord injury (SCI) can cause neurogenic shock accompanied by bradycardia and hypotension. If no preceding traumatic episodes are apparent and the neurological examination is complicated by the patient’s intellectual disability, SCI is likely to be overlooked. A 63-year-old man with intellectual disability presented to our hospital. The patient had fallen on the floor; however, no apparent head or neck trauma was observed. The patient returned home after confirming the absence of intracranial hematoma on computed tomography. However, the patient was re-admitted because of hypotension and bradycardia, and sick sinus syndrome was suspected. As the manifestations were motor weakness in the extremities and urinary retention, screening spinal magnetic resonance imaging revealed cervical cord injury and spondylosis. Cervical SCI related to a fall was suspected. Cervical decompression surgery and rehabilitation therapy contributed to the improved patient status. Herein, we report a case of intellectual disability in which SCI was initially overlooked. No severe preceding traumatic episode or intellectual disability of the patient could have led to overlooking SCI in our case. Clinicians should be cautious about this rare condition.

## Introduction

Spinal cord injuries (SCIs) can be triggered by minor traumatic head and neck accidents, which can lead to neurological deficits; however, they can also result in various clinical manifestations such as bronchopulmonary (67% of acute SCI may experience within the first days post-injury), cardiovascular (if it occurs, the incidence peak maybe four days post-injury), gastrointestinal (27%-62% of patients with SCI may experience), sexual, thermoregulatory (if it occurs, it may appear in the acute phase of SCI and can last a lifetime), and urinary complications [1,2]. Neurogenic shock with hypotension and bradycardia can manifest in SCI [1]. In case any preceding traumatic events are not apparent, clinicians can diagnose patients’ diseases based only on the apparent manifestations secondary to SCI, which can delay the diagnosis and management of SCI.

In particular, when patients have an underlying disease, such as intellectual or developmental disabilities, the neurological examination can be complicated compared to that of patients without those underlying diseases [3]. Therefore, neurological deficits secondary to SCI cannot be overlooked in patients with intellectual or developmental disabilities.

Here, we present a clinically important case of intellectual disability in which SCI after a minor head trauma was initially overlooked, highlighting the difficulty in appropriately diagnosing SCI in those patients with intellectual disability.

## Case presentation

**Figure 1:**
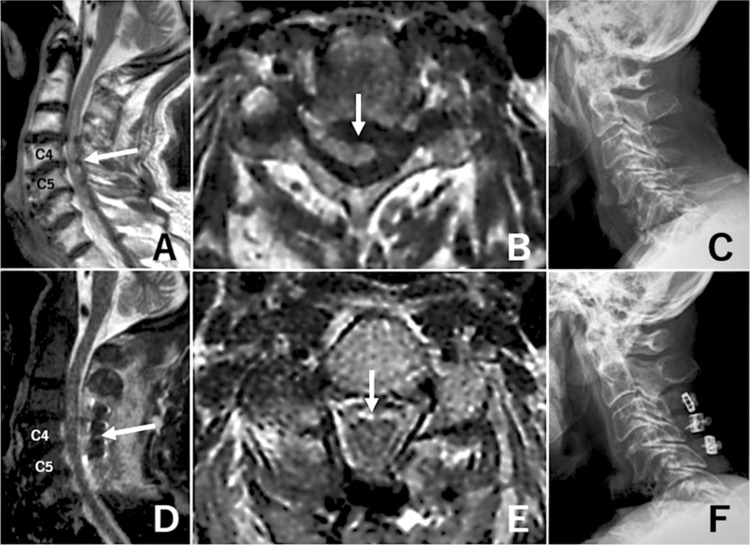
Magnetic resonance images (MRI) and X-ray photographs (A-C) before and (D-F) after surgery. T2-weighted MRIs showing severe cervical cord compression at the C3/4, C4/5, C5/6, and C6/7 levels with intracordal hyperintensity changes. White arrows in (A) sagittal view and (B) axial view showing severe cord compression at the C4/5 level. (C) Lateral radiograph showing cervical spondylosis. T2-weighted short-tau inversion recovery MRIs showing released cervical cord compression. White arrows in (D) sagittal view and (E) axial view showing released cervical cord compression at the C4/5 level. Lateral radiograph showing implantation at the levels of C3, C4, and C5 and removal of the C6 lamina (F).

A 63-year-old man visited our hospital with a member of the nursing care staff. He had lived in a group home for 30 years because he needed support for activities of daily living due to intellectual disability (Intelligence Quotient of 40; extremely low by the Wechsler IQ classification). The patient visited the hospital after experiencing a facial injury following a fall from the floor. He was only taking medications for diabetes mellitus and hypertension. Initially, neurological examination was difficult because the patient did not respond appropriately owing to intellectual disability. Computed tomography (CT) examination did not reveal any intracranial traumatic lesions, and the patient returned home. However, 10 hours after the first visit to our hospital, he was transported to our emergency room because of deteriorated consciousness and unmeasurable blood pressure. Upon arrival, he was drowsy, although he could follow simple instructions. The blood pressure was 80/30 mmHg, and the heart rate was 30 beats per minute (bpm). A cardiologist suspected sick sinus syndrome. An intravenous injection of atropine elevated his heart rate and systolic blood pressure to 70 bpm and 90 mmHg, respectively. However, the effectiveness of atropine was temporary, and permanent pacemaker implantation was considered. Although the medical staff at our hospital were unfamiliar with the usual behavior of the patient, neurological deficits were not apparent. Several days after admission, his heart rate temporarily decreased to 30 bpm several times a day, and spontaneously recovered to 90 bpm without any drugs. As his systolic blood pressure did not decrease below 90 mmHg, he was permitted to leave his bed on admission day 6. As the patient’s status improved, permanent pacemaker implantation was suspended. During the bed rest period, the patient was completely dependent on others for activities of daily living. When he attempted to move into a wheelchair, he was unable to stand independently because of motor weakness in the lower extremities (Manual Muscle Testing of 3 in his lower extremities). Although the patient was able to raise the upper extremities bilaterally, motor weakness was observed (Manual Muscle Testing of 3 in his upper extremities). Because disuse syndrome was suspected, rehabilitation therapy was initiated. Since admission, the patient appeared to urinate independently. However, one nurse noticed abdominal swelling in the patient on admission day 7. Abdominal CT revealed hydronephrosis and urinary retention; therefore, a bladder catheter was inserted. On day 10 after admission, fecal incontinence was suspected with continuing anal leakage. As bladder and rectal disturbances were suspected, whole-spine magnetic resonance imaging (MRI) was performed. It shows intracordal hyperintensity on T2 weighted imaging and severe spinal canal stenosis at C3, C4, C5, and C6 (Figures 1A-1C). These findings suggest cervical spondylosis and SCI related to a preceding traumatic episode. The patient was examined by a neurosurgeon. The symptoms corresponded to the American Spinal Injury Association (ASIA) grade A. Decompression of the cervical cord was necessary. Because his heart rate remained unstable, a temporary pacemaker was inserted. Subsequently, laminectomy of C3 and laminoplasty of C4 to C6 were performed (Figures 1D-1F).

His postoperative course was uneventful, and his heart rate stabilized at >60 bpm without any treatment. The pacemaker was removed on postoperative day 2. The patient continued rehabilitation and recovered to ASIA grade C five months after surgery.

## Discussion

Herein, we present a case of SCI in a patient with intellectual disability. In our case, treatment for hypotension and bradycardia, which were suspected to be sick sinus syndrome, was initiated. However, after motor weakness in the upper and lower extremities and bladder and rectal dysfunction were observed, a spinal lesion was identified. Traumatic SCI was diagnosed based on the MRI findings, and the patient underwent cervical decompression surgery and rehabilitation therapy. SCI was initially overlooked, as neurological examination of the patient was difficult because of unfamiliar communication between the patient with intellectual disability and the medical staff in our hospital. Our case highlights the importance of alerting clinicians to the appropriate diagnosis of SCI in patients with intellectual disability.

The disadvantages of patients with intellectual disability, such as reduced quality of life related to poor health conditions and inappropriate management of undiagnosed diseases, have been reported [4]. The difficulty in accurately evaluating progressive functional decline in patients with intellectual disability has been emphasized because these patients may not be able to convey their symptoms even to familiar caregivers [3]. We supposed that medical interviews and physical tests could be complicated in these patients because they might not respond appropriately through verbal channels. In addition, subtle conditional changes in these patients cannot be easily identified without familiar care staff because the background is unique to each individual. In the present case, after the patient was admitted, he was examined without the support of familiar caregivers. The medical staff were blinded to the patient’s usual condition. Therefore, it is difficult to clinically judge the neurological significance of the patient’s condition. These conditions may have resulted in a delayed diagnosis of SCI.

The acute phase of SCI, defined as 2-48 hours after injury, can cause neurogenic shock [1]. Damage to the autonomic nervous system can result in cardiovascular complications, such as bradycardia and arterial hypotension [1,2,5,6]. Specifically, SCI in the cervical regions is frequently accompanied by those symptoms [1,2,5,6]. The severity of hypotension or bradycardia might depend on the severity of the spinal injury and the injured spinal levels [1,7]. Since the traumatic episode did not seem severe in our patient and no apparent intracranial lesions were identified on the preceding CT images, the possibility that bradycardia and arterial hypotension could have resulted from SCI was not considered. Physicians are generally unfamiliar with the manifestations of SCI. Therefore, the SCI diagnosis was delayed.

Caregivers of a patient with intellectual disability should emphasize the changes in the patient’s activity of daily living or behavior, to be judged the significance of symptoms by a physician. Physicians should be aware that minor traumatic head and neck accidents can trigger SCI. Physician awareness and caregiver education mentioned earlier might help early diagnosis of SCI.

## Conclusions

We describe a clinically important case of intellectual disability in which SCI was initially overlooked. This occurred possibly because no severe preceding head or neck trauma was apparent and because the neurological examination was complicated due to the patient’s intellectual disability. With any severity of head trauma, SCI can occur. Patients with intellectual disabilities might not respond appropriately through verbal channels. Clinicians should be aware of this rare condition to avoid overlooking SCI.
